# 53BP1: keep an eye on merotely

**DOI:** 10.18632/oncotarget.16984

**Published:** 2017-04-09

**Authors:** Mengfan Tang, Junjie Chen

**Affiliations:** Department of Experimental Radiation Oncology, Division of Radiation Oncology, The University of Texas MD Anderson Cancer Center, Houston, TX, USA

**Keywords:** 53BP1, Aurora B, chromosome segregation, merotely, aneuploidy

News on: Aurora kinase B dependent phosphorylation of 53BP1 is required for resolving merotelic kinetochore-microtubule attachment errors during mitosis by Wang, et al. Oncotarget. 2017; 8:48671-48687. https://doi.org/10.18632/oncotarget.16225

p53-binding protein 1 (53BP1) is well known for its roles in DNA damage signaling and DNA repair, especially for coordinating the two major DNA double-strand break (DSB) repair pathways, i.e. it promotes non-homologous end-joining (NHEJ) repair but inhibits homologous recombination (HR) repair [1, 2]. Upon DSB induction in interphase cells, 53BP1 can be recruited to DSB sites via an ATM-mediated signaling cascade, which is initiated by H2AX phosphorylation, then the recruitment of MDC1, RNF8 and RNF168; RNF168 in turn ubiquitylates H2A/H2AX, which triggers the recruitment of 53BP1 [3, 4]. However, cells in mitosis normally inactivate DSB repair to protect chromosomes against telomere fusions [5]. During mitosis, CDK1 and PLK1 mediated phosphorylation of 53BP1 at T1609 and S1618 sites inhibit 53BP1 recruitment to DSB-flanking chromatin, restoration of 53BP1 accumulation at mitotic DSB sites reactivates DNA repair but leads to aberrant telomere fusions [5]. Although significant progress towards 53BP1 function in DNA damage response has been made, an interesting early observation that 53BP1 can tightly binds to outer kinetochores during mitosis in unstressed cells [6] has never been fully investigated. A report by Hegde and colleagues in this issue of Oncotarget (Wang et al.) provides new insight of 53BP1 in resolving merotelic kinetochore-microtubule attachments during mitosis, which is independent of its function in DNA damage response but acts in an Aurora B kinase activity-dependent manner.

Wang et al observed that in unstressed cells, 53BP1 association with kinetochore is dynamically regulated, since it appears on kinetochores at prophase, is partially retained until metaphase, and then dissociates during anaphase. 53BP1 is phosphorylated by Aurora B at serine1342 and this phosphorylation is required for the optimal recruitment of 53BP1 to kinetochores. Inactivation of Aurora B kinase activity or mutation of 53BP1 serine1342 to alanine did not affect 53BP1 foci formation after IR and these cells displayed normal DSB repair kinetics when compared with control cells, indicating that phosphorylation of 53BP1 at S1342 is not involved in DNA damage response.

To further study the mitotic function of 53BP1, the authors utilized U2OS cell line stably expressing EGFP-histone-H2B to visualize chromosome dynamics and the timing of mitotic transitions by time-lapse microscopy. They found that 53BP1-depleted anaphase cells showed significant accumulation of lagging chromosomes together with defects in resolving spontaneously formed chromosome bridges.

In response to certain kinetochore-microtubule attachment errors, (e.g. syntelic), mammalian cells activate the spindle assembly checkpoint (SAC) to arrest cells at pro-metaphase to prevent lagging chromosomes [7]. However, 53BP1-depleted cells showed no change in pro-metaphase duration. Wang et al then analyzed 53BP1-associated proteins in mitosis and identified the mitosis-dependent interaction of 53BP1 and MCAK. MCAK is a microtubule depolymerase that depolymerize improperly attached microtubules at the kinetochore to ensure accurate chromosome segregation [8]. Wang et al proposed that 53BP1 may be involved in merotelic kinetochore orientation. This hypothesis was clearly supported by the observations that 53BP1 depleted anaphase cells showed lagging chromosomes with highly distorted kinetochores together with elevated aneuploidy and that MCAK helped the stabilization of 53BP1 on merotelically-attached kinetochores during mitosis.

This new insight into 53BP1 mitotic function indicates that 53BP1 should at least play two important roles in mitotic surveillance: on one hand 53BP1 is excluded from mitotic DSB sites to help inactive DSB repair at mitotic cells to guard against telomere fusions upon DNA damage; on the other hand, 53BP1 keeps an eye on spontaneous kinetochore-microtubule attachment errors (merotely) to ensure chromosomal stability. Precise regulation of kinetochore-microtubule attachment and chromosome segregation is critical for the maintenance of genomic stability in which defective in these processes may linked to tumor progression as well as resistance to cancer therapy in cells with 53BP1 deficiency.
